# Characterization of central venous stenosis induced by indwelling catheter and balloon injury in a rabbit model: angiographic, histological, and molecular evaluations

**DOI:** 10.1186/s42155-026-00700-z

**Published:** 2026-05-29

**Authors:** Jian Huang, Ke Li, Hui Yuan, Dayong Zhou, Xicheng Zhang

**Affiliations:** 1 The Fourth Affiliated Hospital of Soochow University, Suzhou, Jiangsu China; 2 The Affiliated Suzhou Hospital of Nanjing Medical University, Suzhou Municipal Hospital, No. 26 Daoqian Street, Suzhou, Jiangsu China

## Abstract

**Background:**

Central venous stenosis (CVS) is a significant complication in patients undergoing hemodialysis. Although mechanical injury and foreign body reactions are known triggers, the differential pathophysiological mechanisms underlying CVS induced by transient balloon injury and chronic indwelling catheters remain unclear. This study compared stenotic phenotypes and molecular pathways in rabbit models of balloon-induced (Ap) and catheter-related (Ac) CVS.

**Methods:**

Rabbit models were established using balloon dilation (Ap group, *n* = 9) or indwelling catheter implantation (Ac group, *n* = 10) in the anterior vena cava. Vessel patency was assessed using serial angiography on day 30. Histomorphological changes were quantified using HE and Masson’s trichrome staining. The molecular profile of vascular remodeling was characterized by immunohistochemical analysis of α-SMA, TGF-β1, MMP-2, and CD31.

**Results:**

Angiography revealed a disparity in stenosis incidence and severity between the two groups. The Ac group exhibited a 100% incidence with severe luminal narrowing (median stenosis rate: 66.5%), whereas the Ap group showed a lower incidence (33.3%) and mild heterogeneous stenosis (median: 3.5%). Histologically, the Ac group demonstrated a 3.5-fold increase in intimal thickness (416.7 ± 10.8 μm vs. 119.7 ± 7.2 μm, *P* < 0.01) and extensive collagen deposition (41.7% vs. 14.7%, *P* < 0.01). The Ac group was characterized by fibrin sheath formation and significantly upregulated expression of profibrotic and remodeling markers (α-SMA, TGF-β1, MMP-2, and CD31).

**Conclusion:**

Indwelling catheters induce a more severe and uniform stenotic phenotype than transient mechanical injury, owing to exacerbated fibrosis and vascular remodeling pathways involving TGF-β1 and MMP-2. Hence, the pathophysiology of catheter-related CVS differs from that of balloon-induced injury, highlighting the need for targeted antifibrotic therapies for catheter-related complications.

## Introduction

Central venous catheters (CVCs) are essential for patients with end-stage renal disease (ESRD), especially those initiating hemodialysis or awaiting arteriovenous fistula maturation [6]. However, prolonged CVC use often leads to CVS, a serious complication that compromises vascular access patency and significantly increases patient morbidity [7]. Clinically, CVS often manifests as symptomatic venous hypertension, causing ipsilateral limb edema, pain, and collateral vein formation. This can render the affected limb unsuitable for future vascular access creation. The high-flow state of a mature AVF converts compensated stenosis into a high-resistance outflow obstruction, leading to severe venous hypertension, ipsilateral limb edema, and early AVF failure [4]. CVS critically affects AVF outcomes. In patients undergoing AVF creation, pre-existing or catheter-induced CVS can precipitate early AVF failure by exacerbating venous hypertension, reducing outflow capacity, and promoting intimal hyperplasia, thereby directly undermining the success and long-term patency of the vascular access [2]. Despite its high prevalence and clinical burden, the precise pathophysiological mechanisms driving catheter-related CVS remain unclear, and effective therapeutic strategies are lacking [1].

The current understanding of CVS pathophysiology largely stems from animal models of vascular injury. The balloon injury model is the most common experimental platform for studying neointimal hyperplasia [10]. Although this model effectively replicates acute endothelial denudation and mechanical stretch associated with angioplasty, it represents a transient, “one-hit” mechanical trauma [3]. Contrastingly, clinical catheter-related CVS is more complex, involving chronic, continuous mechanical irritation and a persistent foreign body reaction. Furthermore, most existing models evaluate CVS in a low-flow environment and fail to consider the synergistic effect of the high-flow hemodynamic characteristics of AVF, which is the primary clinical context in which CVS becomes symptomatic [5, 15]. Consequently, the standard balloon injury model may fail to capture the complex inflammatory and fibrotic processes unique to catheter-induced lesions, limiting the translational value of findings derived solely from this model [11, 12].

Therefore, we used a rabbit model that combined an established AVF with central venous injury to elucidate the distinct pathological mechanisms of catheter-related CVS. Two injury modalities, a transient balloon injury model (Ap group) and an indwelling catheter model (Ac group), were compared and subjected to the high-flow conditions of a functional AVF. We hypothesized that the chronic presence of a catheter induces a more severe stenotic phenotype driven by aggravated fibrosis and sustained vascular remodeling than transient mechanical injury alone. By systematically evaluating angiographic patency, histomorphological changes, and transforming growth factor-beta1 (TGF-β1), alpha-smooth muscle actin (α-SMA), matrix metalloproteinase-2 (MMP-2), we aimed to characterize the differential contributions of acute trauma versus chronic foreign body stimulation in the pathogenesis of CVS, thereby providing a more clinically relevant experimental basis for preserving AVF function.

## Materials and methods

### Ethical statement and animal grouping

This study was conducted in strict accordance with the *Guide for the Care and Use of Laboratory Animals*. The experimental protocol was approved by the Institutional Animal Care and Use Committee (IACUC) of the Fourth Affiliated Hospital of Soochow University. Twenty New Zealand white rabbits (initial weight 2.5–3.0 kg) were obtained and housed under standard conditions. The rabbits were randomly divided into two groups (*n* = 10 per group, One rabbit in the Ap group was excluded due to acute thrombosis post-surgery, resulting in *n*=9 for the final analysis): the balloon injury group (Ap group) and the catheter implantation group (Ac group).

### Anesthesia and preoperative preparation

Anesthesia was induced at least 30 min prior to surgery. A mixture of tiletamine hydrochloride and zolazepam hydrochloride (3 mg/kg) was administered intravenously via the marginal ear vein, followed by an intramuscular injection of Sumianxin II (0.02 mL/kg) into the hind limb. Rabbits were placed in a supine position on a fixation table, and their limbs and incisors were secured. The cervical area was shaved and disinfected. Preoperative central venous angiography was performed by injecting iodixanol contrast agent via the marginal ear vein to confirm baseline vascular anatomy.

### Surgical procedures

**Fig. 1:**
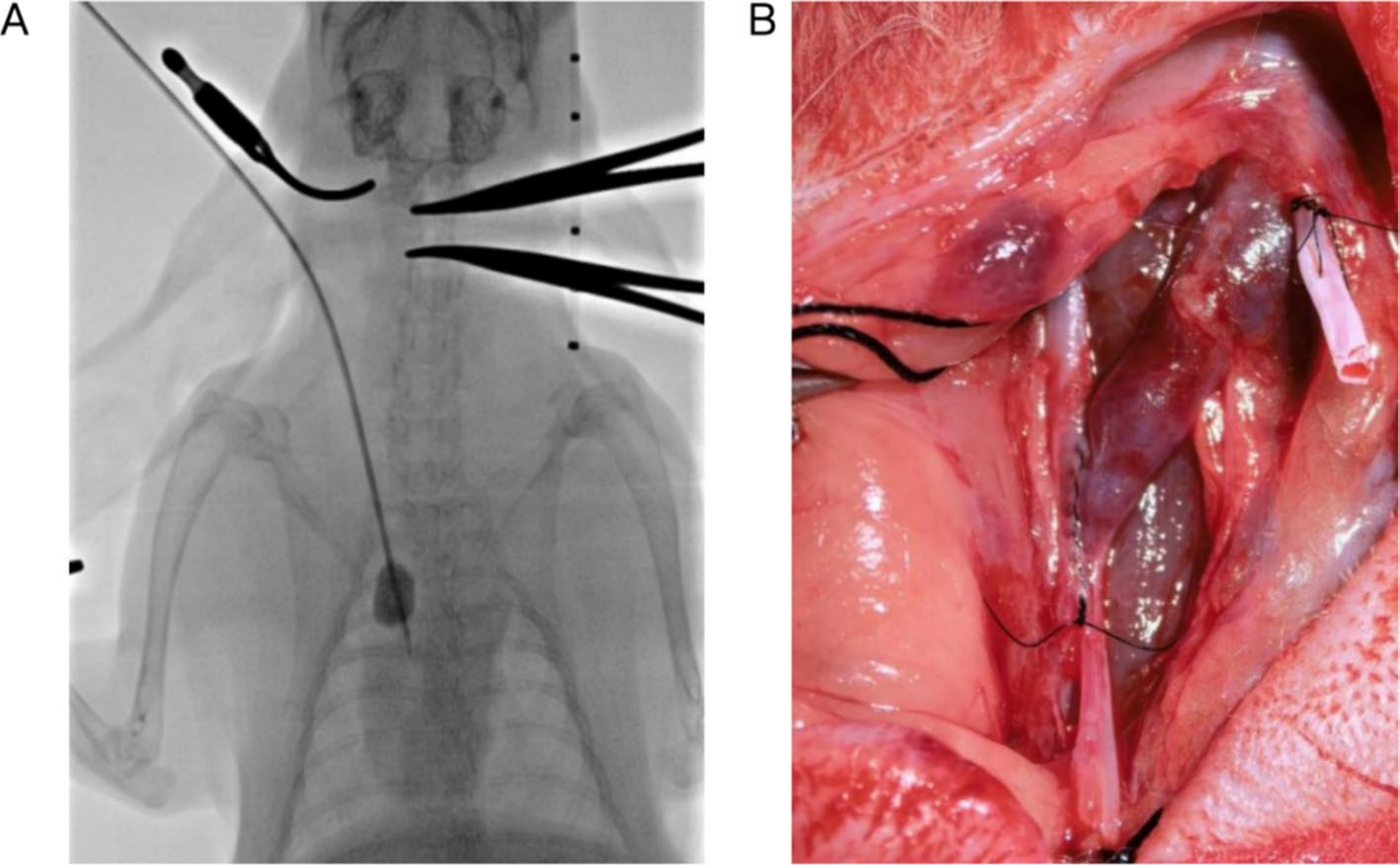
Surgical procedures for model establishment. **A** Balloon injury model (Ap group): The anterior vena cava was dilated using a 5.5F Fogarty balloon. **B** Indwelling catheter model (Ac group): A catheter was implanted into the anterior vena cava

All surgical procedures were performed under sterile conditions. The right external jugular vein (EJV) medial branch and the common carotid artery (CCA) were isolated. (1) Balloon Injury Model (Ap group): a 5-mm longitudinal incision was made in the medial branch of the EJV. A 1.83 mm (5.5 Fr) compliant balloon catheter (Fogarty; Edwards Lifesciences, Irvine, CA, USA) was inserted and advanced into the cranial vena cava. To induce standardized mechanical injury, the balloon was inflated to a diameter of 11 mm, achieving a 3.6-fold overstretch injury relative to the native vein diameter (~3.1 mm). The balloon was inflated for 1 min and deflated; this procedure was repeated 5 times to ensure consistent endothelial denudation and medial stretching, as previously described[13]. (2)Catheter Implantation Model (Ac Group): Following the establishment of a side-to-side CCA-EJV AVF, a 0.97 mm (2.9 Fr) silicone central venous catheter (outer diameter: 0.96 mm) was advanced into the cranial vena cava under fluoroscopic guidance. Given the average vein diameter of 3.1 ± 0.3 mm, the catheter occupied approximately 9.5% of the vessel’s cross-sectional area. This size was selected to ensure continuous mechanical contact with the vein wall while remaining non-occlusive, thereby allowing high-flow blood from the AVF to pass through and create the turbulent hemodynamic environment characteristic of clinical CVS. The indwelling catheter was not used for any clinical purpose during the study (Fig. 1).

### Postoperative management

Postoperatively, animals were monitored daily for wound bleeding, infection, dietary intake, activity, and body weight. The patency of the AVF was assessed daily by auscultation of the vascular bruit (thrill) using a stethoscope.

### Angiographic evaluation

On day 28 post-surgery, rabbits were anesthetized using the protocol described above. Central venous angiography was performed via the marginal ear vein using iodixanol. Central venous stenosis (CVS) was defined as a luminal diameter reduction of ≥ 50% compared to the adjacent normal vessel or baseline. The stenosis rate and the success rate of model establishment were recorded.

### Histological analysis

Following angiography, animals were euthanized. The bilateral anterior vena cavas were harvested; the right side served as the experimental sample, and the left side served as the self-control. Tissues were fixed in 10% neutral buffered formalin, embedded in paraffin, and sectioned at 4 μm thickness.Sections were stained with Hematoxylin and Eosin (HE) for morphological evaluation, Masson’s trichrome to assess collagen deposition.Images were captured using an optical microscope. Intimal thickness was measured using Image-Pro Plus 6.0 software (Media Cybernetics, USA), defined as the perpendicular distance from the internal elastic lamina to the luminal surface.

### Immunohistochemistry (IHC)

IHC was performed to evaluate vascular remodeling markers. Sections were deparaffinized, rehydrated, and subjected to antigen retrieval. Endogenous peroxidase activity was blocked. Sections were incubated overnight at 4 °C with primary antibodies against CD31 (1:100, Abcam), α-SMA (1:200, Abcam), MMP-2 (1:100, Abcam), and TGF-β 1 (1:100, Abcam). After washing, sections were incubated with HRP-conjugated secondary antibodies and visualized with Diaminobenzidine chromogen. The integral optical density (IOD) of positive staining was analyzed using Image-Pro Plus 6.0.

### Statistical analysis

Data were analyzed using GraphPad Prism software(version 9.0, GraphPad Software, San Diego, CA, USA). Continuous variables are presented as mean ± standard deviation (SD) or median (range) depending on data distribution. Comparisons between two groups were performed using the Student’s t-test for normally distributed data or the Mann-Whitney U test for non-normally distributed data. A *P*-value < 0.05 was considered statistically significant.

## Results

### Establishment of central venous stenosis models and angiographic evaluation

To evaluate central venous stenosis (CVS) formation, two rabbit models were established; a balloon injury model (Ap group) and an indwelling catheter model (Ac group). Angiography assessed vessel patency. In the Ap group (*n*=9), angiographic outcomes showed significant heterogeneity. The incidence of central venous stenosis was 33.3% (3/9 rabbits). Most animals (6/9) exhibited negligible narrowing (<5%), indicating successful vascular repair, whereas three cases developed stenosis ranging from 25 to 78%. The median stenosis rate in the Ap group was 3.5% (range: 0–78%).

**Fig. 2:**
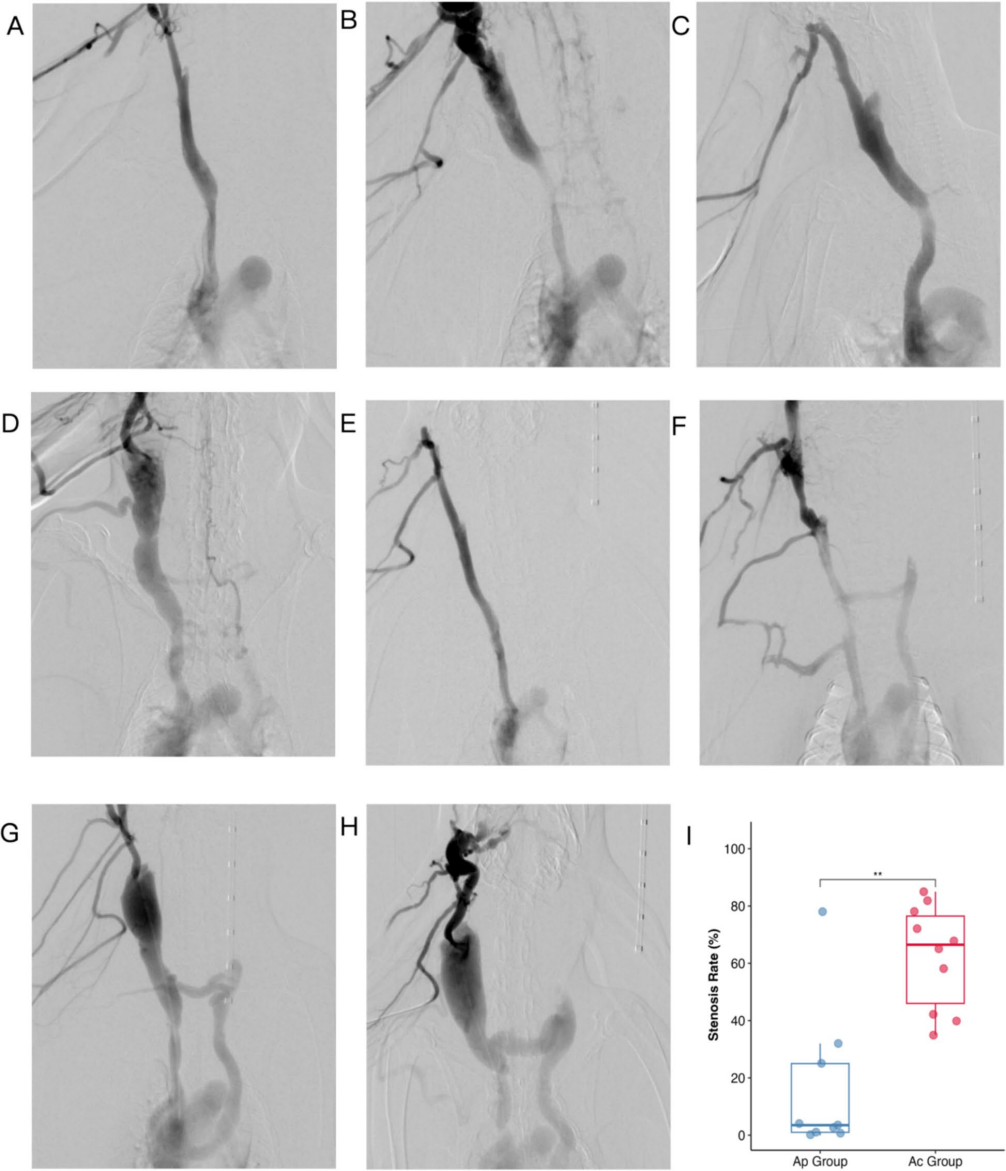
Angiographic evaluation of central venous stenosis. **A**, **B** Representative angiograms of the Ap group before and immediately after balloon dilation. **C** Ap group at 30 days post-surgery, showing no significant stenosis of the anterior vena cava. **D** Ap group at 30 days post-surgery, showing mild stenosis. **E**–**F** Representative angiograms of the Ac group before and immediately after catheter implantation. **G** Ac group at 30 days post-surgery, showing moderate stenosis. **H** Ac group at 30 days post-surgery, showing severe stenosis. **I** Quantitative analysis of angiographic stenosis rates at day 30. Data are presented as box plots (**P < 0.01)

Strikingly, the Ac group (*n*=10) exhibited 100% incidence of stenosis. The degree of narrowing was consistently high (median stenosis rate: 66.5% [35–85%]). Notably, 70% of the animals in the Ac group developed moderate-to-severe stenosis. Quantitative analysis confirmed that the angiographic stenosis rate in the Ac group was significantly higher than that in the Ap group (Fig. 2I, *P* < 0.01).

### Histopathological characterization of intimal hyperplasia

Hematoxylin and eosin (HE) staining quantified vascular remodeling. The Ap group showed variable intimal proliferation, consistent with the angiographic findings, with intimal thickness ranged from 65 to 340 μm (mean: 145.6  ±  85.2 μm). The intima-to-media (I/M) ratio in the Ap group was 1.26 ± 0.6.

**Fig. 3:**
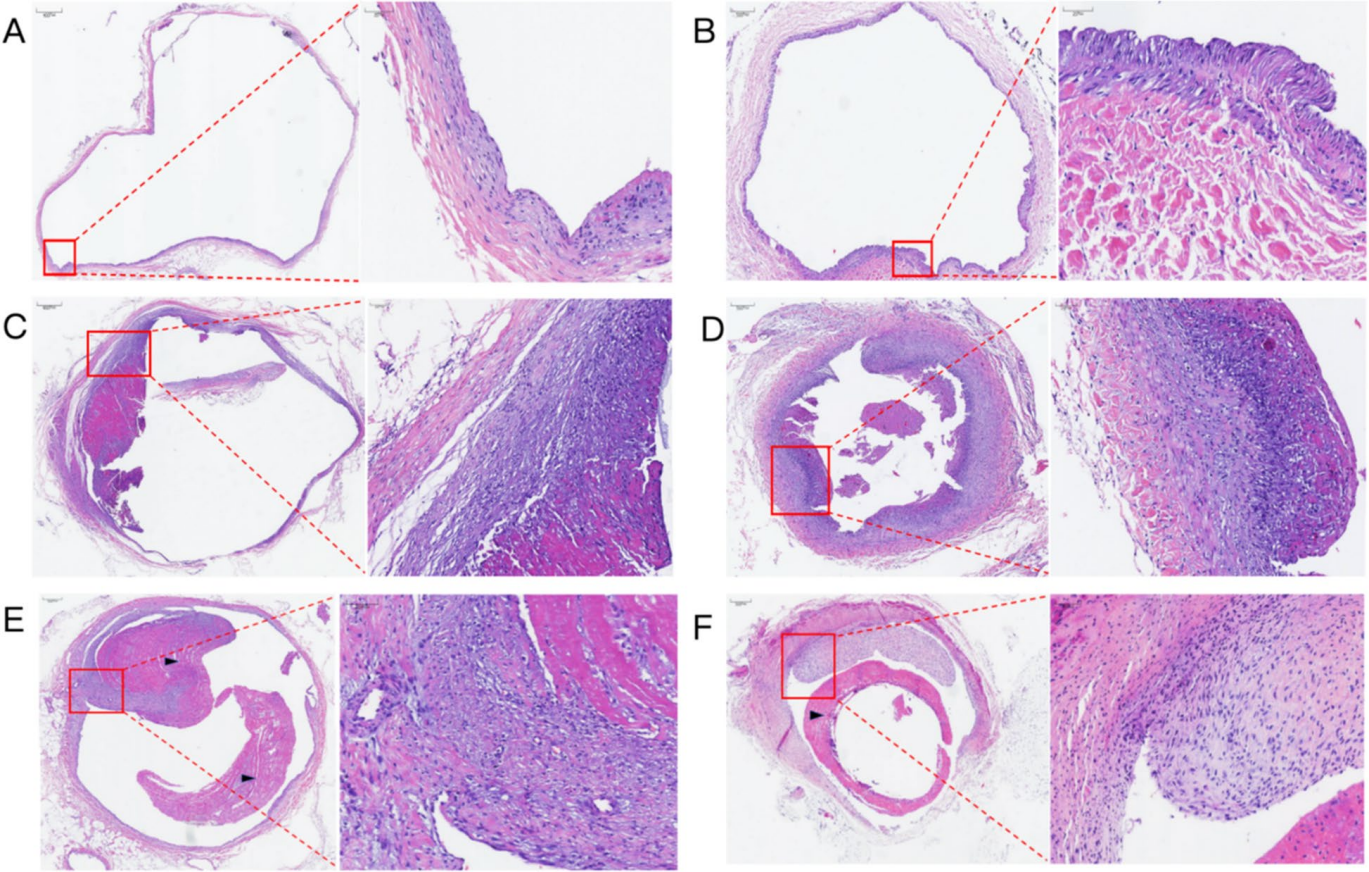
Histopathological evaluation of the anterior vena cava. **A** Normal contralateral anterior vena cava (Control). **B** Significant intimal thickening accompanied by focal neointimal hyperplasia. **C** Representative image of moderate stenosis in Ap group. **D** Representative image of severe stenosis in Ap group. **E**–**F** Representative images of severe stenosis in Ac group. The black triangle (▲) indicates the formation of a fibrin sheath

**Fig. 4:**
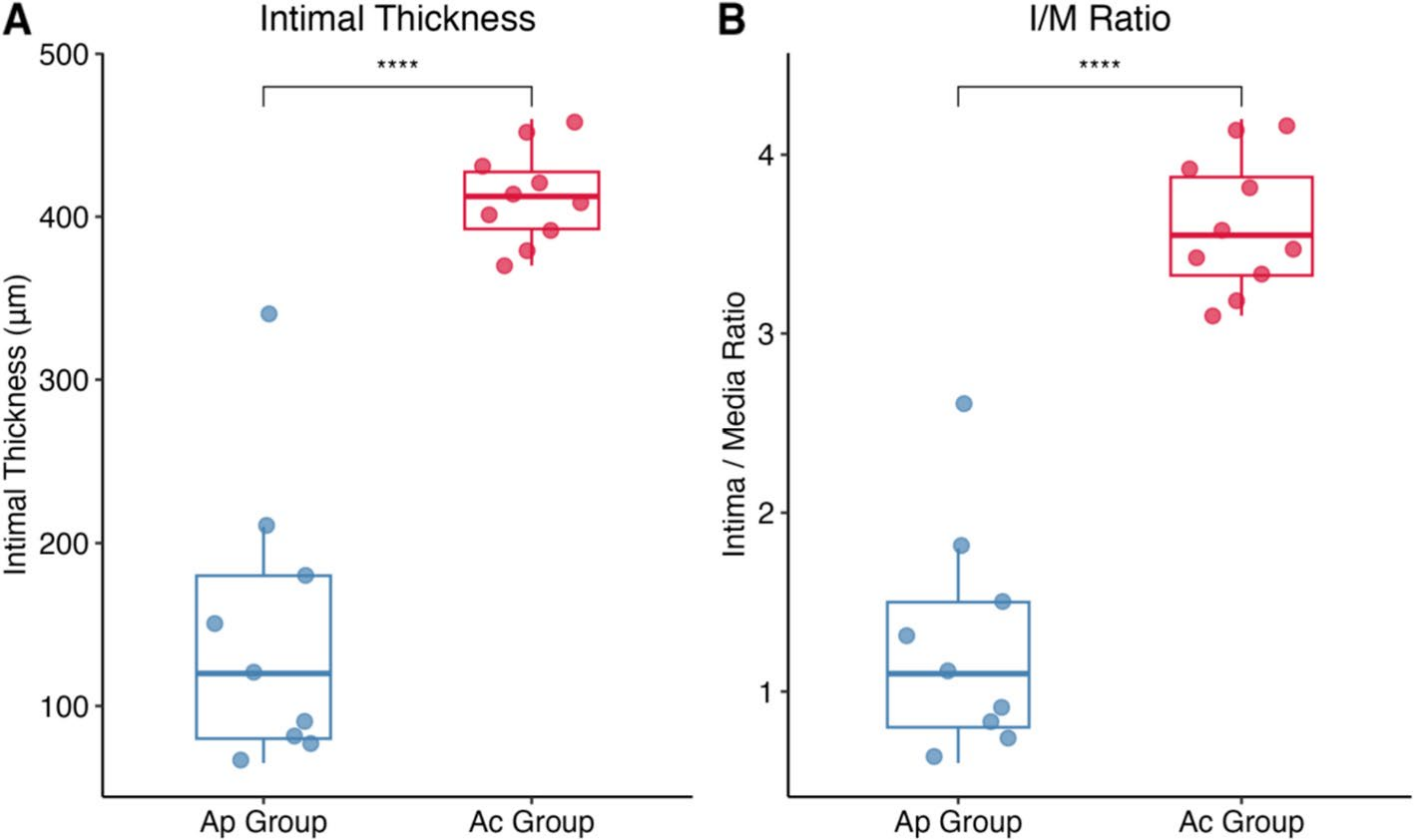
Intimal thickness and intima-to-media ratio. Quantitative analysis of intimal thickness(A) and intima/media (I/M) ratio(B). Data are presented as box plots (*P < 0.05, **P < 0.01)

Contrastingly, the Ac group exhibited severe and uniform pathological changes characterized by extensive neointimal proliferation and luminal obstruction. The mean intimal thickness in the Ac group was 412.5 ± 26.4 μm, approximately 2.8-fold higher than that of the Ap group. The I/M ratio was significantly elevated to 3.61 ± 0.36 (3.1–4.2). Statistical analysis demonstrated that intimal thickness and the I/M ratio were significantly greater in the Ac group than in the Ap group (Figs. 3 and 4A B, *P* < 0.01), indicating that the indwelling catheter induced a more aggressive proliferative response.

### Evaluation of fibrosis and molecular mechanisms of vascular remodeling

Masson’s trichrome staining was used to visualize collagen fibers (stained blue). The Ap group showed mild-to-moderate fibrosis, with a mean collagen area fraction of 14.7%. Contrastingly, the Ac group presented with pronounced fibrosis characterized by dense collagen deposition within the neointima. The mean collagen area fraction in the Ac group increased significantly to 41.7% (Fig. 6A, *P* < 0.01).

**Fig. 5:**
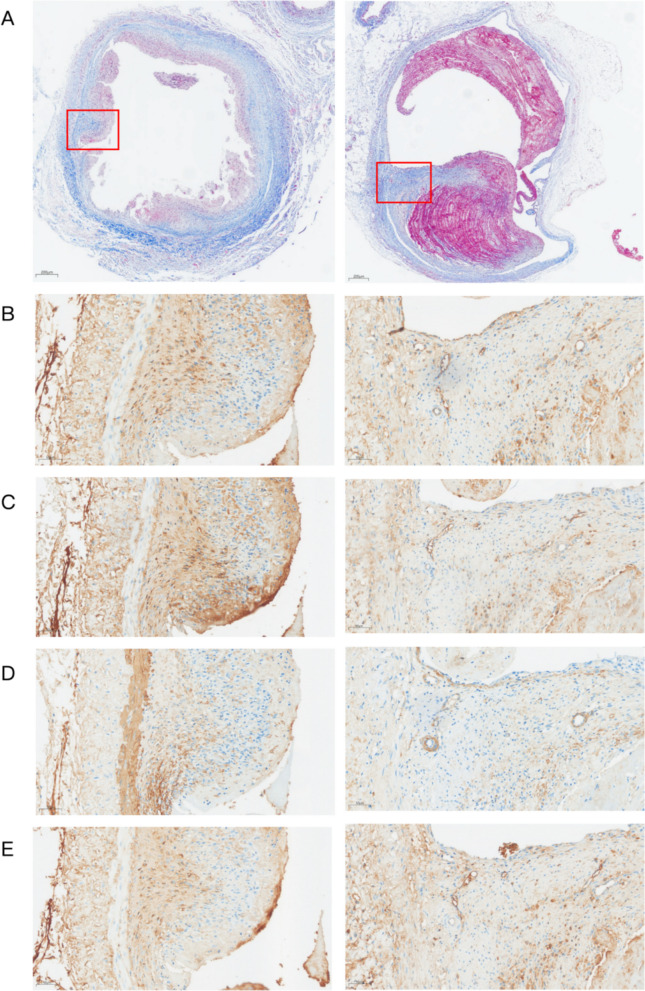
Histological assessment of fibrosis and expression of vascular remodeling markers. **A** Masson's trichrome staining of the anterior vena cava in the Ap group (left) and Ac group (right). Blue staining indicates collagen deposition. **B**-**E** Immunohistochemical analysis of CD31 (**B**), MMP-2 (**C**), α-SMA (**D**), and TGF-β 1 (**E**). The left column displays representative images from the Ap group, and the right column displays images from the Ac group. The Ac group exhibited more pronounced fibrosis and higher expression levels of MMP-2, α-SMA, and TGF-β 1 compared to the Ap group. Scale bar = 50um

**Fig. 6:**
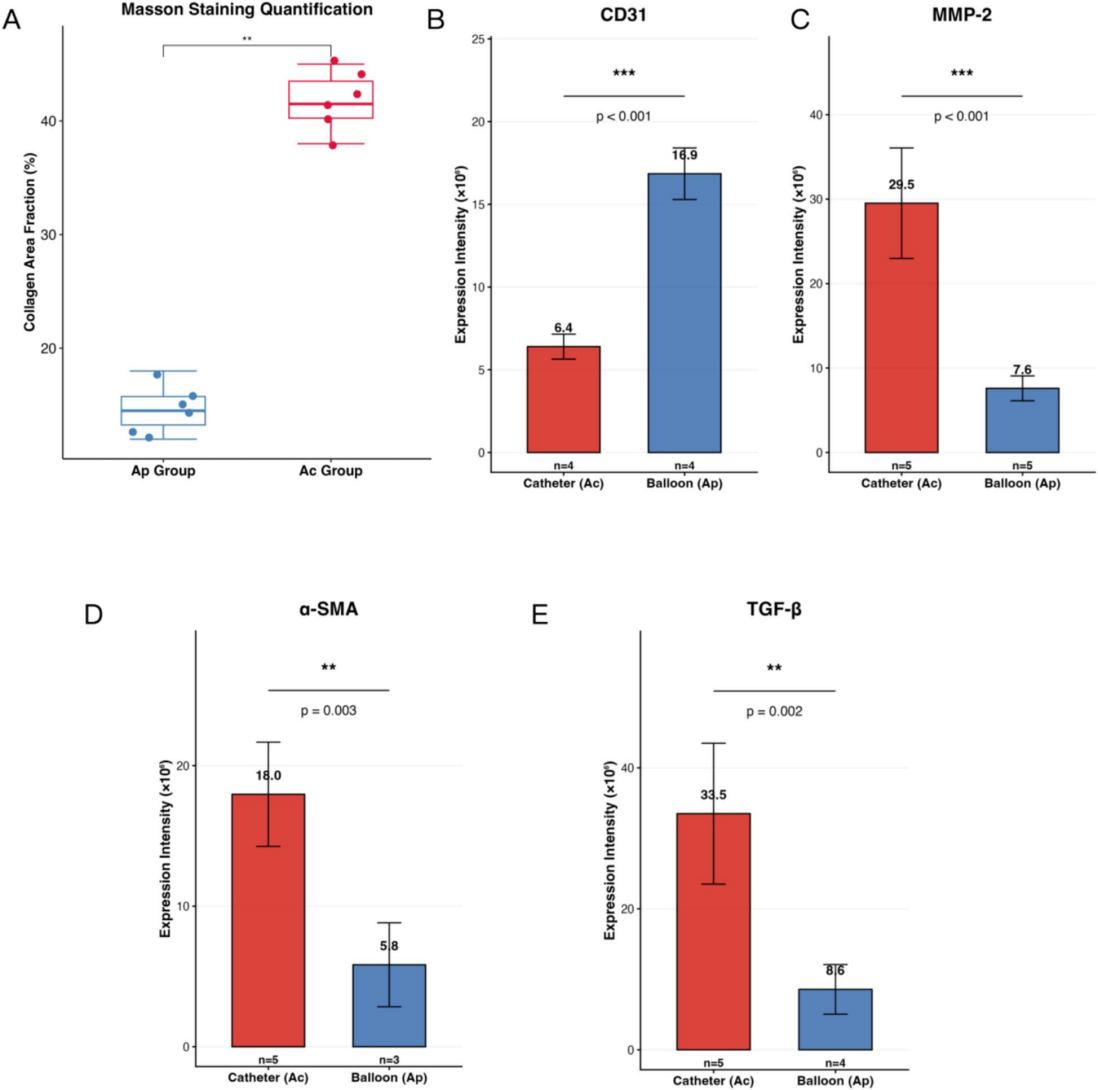
Quantification of collagen and protein markers related to fibrosis and vascularization. Quantitative analysis of the collagen area fraction (**A**) and the integral optical density (IOD) of CD31 (**B**), MMP-2 (**C**), α-SMA (**D**), and TGF-β 1 (**E**). Data are presented as box plots (*P < 0.05, **P < 0.01)

To investigate the molecular mechanisms of stenosis, we assessed the expression of key markers for endothelial integrity, inflammation, and fibrosis via immunohistochemistry (Figs. 5 and 6). The Ap group showed relatively high expression of the endothelial marker CD31 (mean Norm_IntDen: 16.85), suggesting effective re-endothelialization following balloon injury. However, the Ac group showed significantly reduced CD31 expression (mean Norm_IntDen: 6.40, Fig. 6B, *P* < 0.01), indicating impaired endothelial repair and persistent endothelial damage from the indwelling catheter. MMP-2 expression, a key enzyme in matrix degradation and remodeling, was markedly upregulated in the Ac group (mean: 29.52) compared to the Ap group (mean 7.60, Fig. 6C, *P* < 0.01), reflecting active tissue remodeling in the catheterized vessels. α-SMA staining revealed significant accumulation of smooth muscle cells/myofibroblasts in the neointima of the Ac group. The mean intensity in the Ac group was 17.96, approximately 3-fold higher than that in the Ap group (mean: 5.83, Fig. 6D, *P* < 0.01). Consistent with Masson staining, the expression of the profibrotic cytokine TGF-β1 was dramatically elevated in the Ac group (mean: 33.46) compared to the Ap group (mean 8.58, Fig. 6E, *P* < 0.01).

Collectively, these data suggest that the indwelling catheter exacerbates stenosis via sustained endothelial injury (low CD31), followed by aggressive smooth muscle cell proliferation (α-SMA) and fibrosis (TGF-β1/MMP-2).

## Discussion

This study compared two rabbit models of central venous stenosis (CVS) to elucidate the differential effects of acute mechanical injury and chronic indwelling stimulation. Our findings demonstrated a dichotomy in the outcomes of these approaches. Despite using a 1.83 mm (5.5 Fr) balloon (about 1–2 atm) to achieve a significant 3.6-fold overstretch injury, the balloon injury (Ap) model yielded a low incidence of stenosis (33.3%) and significant heterogeneity in the venous system. Contrastingly, the indwelling catheter (Ac) model successfully recapitulated the clinical features of severe CVS, achieving a 100% stenosis rate characterized by extensive neointimal hyperplasia, robust fibrosis, and fibrin sheath formation. This suggests that chronic foreign body stimulation, although non-occlusive (occupying approximately 9.5% of the lumen), drives a more aggressive vascular remodeling program than transient mechanical trauma.

The fundamental difference in stenosis severity between the two groups likely stems from distinct pathophysiological mechanisms triggered by “single-hit” versus “continuous” insults. In the Ap group, balloon dilatation caused immediate endothelial denudation and medial stretching. Although the pressure used is lower than clinical high-pressure angioplasty (20–40 atm), the resulting 3.6:1 stretch ratio represents severe mechanical trauma to the delicate rabbit vein. However, in the high-flow, low-pressure environment of the vena cava, this acute injury often resolves through reendothelialization, which explains the high rate of spontaneous patency observed. The 0.97 mm (2.9 Fr) catheter creates a constant source of friction and flow disturbance without completely occluding the vessel, which is essential for maintaining a high-flow state provided by the concomitant AVF.

A unique feature of our model is the simultaneous creation of an AVF that significantly altered the hemodynamic landscape. Clinical CVS often develops before the AVF is present. However, we established a high-flow AVF to simulate a clinical scenario in which CVS becomes symptomatic. By performing these procedures in a single surgical step, the early injury-repair response of the venous wall coincides with physiological shifts in AVF maturation. Maturation of the AVF increases venous blood flow and shear stress, likely synergizing with catheter-related inflammation to accelerate neointimal hyperplasia. This “high-flow + chronic irritation” environment more accurately reflects the “ticking time bomb” effect observed in patients with ESRD, in which latent stenosis is unmasked and exacerbated by creating a permanent access [7].

The indwelling catheter acts as a persistent nidus for inflammation. Continuous mechanical friction and the foreign materials likely sustain a local inflammatory milieu, preventing wound healing resolution. This chronic irritation is evidenced by the significant upregulation of TGF-β1 in the Ac group. TGF-β1 is crucial in promoting fibrosis and stenosis in hemodialysis access dysfunction [8]. Sustained TGF-β1 signaling promotes the transition of fibroblasts and smooth muscle cells into a synthetic, proliferative myofibroblast phenotype, confirmed by the intense α-SMA expression. Concurrently, elevated MMP-2 levels suggest active, albeit dysregulated, turnover of the extracellular matrix, facilitating cell migration and collagen accumulation (as observed by Masson staining). Additionally, the fibrin sheath observed in the Ac group may have served as a provisional scaffold, allowing endothelial cells (as indicated by CD31) and myofibroblasts to migrate along the catheter surface, thereby accelerating luminal narrowing. This mechanism is supported by clinical imaging studies that correlate fibrin sheath formation with catheter-related stenosis [9].

Our findings have significant translational implications in the study and treatment of catheter-related thrombosis and stenosis. First, the high failure rate of the balloon injury model suggests that it is an inadequate tool for studying CVS as it fails to mimic the chronic inflammatory component of indwelling devices. Researchers aiming to test novel stents or drug-coated balloons for venous pathologies should prioritize the indwelling catheter model as it more accurately reflects the recalcitrant nature of clinical lesions. Second, the identification of the fibrin sheath and TGF-β1-mediated fibrotic pathway highlights potential therapeutic targets. Current treatments rely heavily on mechanical angioplasty, which often fails because of elastic recoil and restenosis. Our data suggest that pharmacological interventions targeting the TGF-β1 signaling pathway or inhibiting myofibroblast differentiation, delivered via drug-eluting technologies, could be promising strategies for preventing the progression of catheter-induced stenosis. This approach is supported by growing preclinical evidence for anti-fibrotic therapies in vascular remodeling [14].

This study has several limitations. First, the balloon pressure, although appropriate for the rabbit anatomy to avoid rupture, does not fully replicate the ultrahigh pressures used in clinical human angioplasty. Second, the simultaneous creation of AVF and venous injury may confound the isolated effects of the catheter; however, this design was selected because of its high clinical relevance to the hemodynamics of patients with ESRD. Finally, although we identified correlations between stenosis severity and markers, this study was observational. Future studies utilizing specific inhibitors or knockout models are warranted to establish a definitive causal link between these molecular pathways and venous remodeling.

Overall, this study demonstrated that the indwelling catheter model induced more severe, consistent, and fibrotic central venous stenosis compared to the balloon injury model. Although balloon injury results in heterogeneous stenosis rates with mild intimal hyperplasia, the chronic mechanical irritation and inflammation caused by the indwelling catheter led to a 100% incidence of stenosis, characterized by extensive neointimal hyperplasia, significant collagen deposition, and the upregulation of profibrotic markers. These findings suggest that the indwelling catheter model replicates the complex pathophysiology of catheter-related central venous stenosis observed in clinical practice. Therefore, this model serves as a superior platform for investigating the underlying mechanisms of CVS and evaluating potential therapeutic interventions.

## Data Availability

I confirm I have included a data availability statement in my main manuscript file. My manuscript has associated data in a data repository.
